# Broad Impairment of Natural Killer Cells From Operationally Tolerant Kidney Transplanted Patients

**DOI:** 10.3389/fimmu.2017.01721

**Published:** 2017-12-11

**Authors:** Emilie Dugast, Gaëlle David, Romain Oger, Richard Danger, Jean-Paul Judor, Katia Gagne, Mélanie Chesneau, Nicolas Degauque, Jean-Paul Soulillou, Pascale Paul, Christophe Picard, Pierrick Guerif, Sophie Conchon, Magali Giral, Nadine Gervois, Christelle Retière, Sophie Brouard

**Affiliations:** ^1^Centre de Recherche en Transplantation et Immunologie UMR1064, INSERM, Université de Nantes, Nantes, France; ^2^Institut de Transplantation Urologie Néphrologie (ITUN), CHU Nantes, Nantes, France; ^3^Etablissement Français du sang, Nantes, France; ^4^CRCINA, INSERM, Université d’Angers, Université de Nantes, Nantes, France; ^5^LabEx Transplantex, Université de Strasbourg, France; ^6^Nephrology Dialysis Renal Transplantation Center, Assistance Publique des Hôpitaux de Marseille, Hospital de la Conception, UMR 1076, Vascular Research Center of Marseille, INSERM, Aix-Marseille University, Marseille, France; ^7^Établissement Français du Sang Alpes Méditerranée, Marseille, France; ^8^ADES UMR 7268, CNRS, EFS, Aix-Marseille Université, Marseille, France; ^9^CIC Biotherapy, CHU Nantes, Nantes, France

**Keywords:** natural killer, cytotoxicity, tolerance, kidney, transplantation

## Abstract

The role of natural killer (NK) cells in organ transplantation is controversial. This study aims to decipher their role in kidney transplant tolerance in humans. Previous studies highlighted several modulated genes involved in NK cell biology in blood from spontaneously operationally tolerant patients (TOLs; drug-free kidney-transplanted recipients with stable graft function). We performed a phenotypic, functional, and genetic characterization of NK cells from these patients compared to kidney-transplanted patients with stable graft function under immunosuppression and healthy volunteers (HVs). Both operationally TOLs and stable patients harbored defective expression of the NKp46 activator receptor and lytic molecules perforin and granzyme compared to HVs. Surprisingly, NK cells from operationally TOLs also displayed decreased expression of the CD16 activating marker (in the CD56^Dim^ NK cell subset). This decrease was associated with impairment of their functional capacities upon stimulation, as shown by lower interferon gamma (IFNγ) production and CD107a membranous expression in a reverse antibody-dependent cellular cytotoxicity (ADCC) assay, spontaneous lysis assays, and lower target cell lysis in the ^51^Cr release assay compared to HVs. Conversely, despite impaired K562 cell lysis in the ^51^Cr release assay, patients with stable graft function harbored a normal reverse ADCC and even increased amounts of IFNγ^+^ NK cells in the spontaneous lysis assay. Altogether, the strong impairment of the phenotype and functional cytotoxic capacities of NK cells in operationally TOLs may accord with the establishment of a pro-tolerogenic environment, despite remaining highly activated after transplantation in patients with stable graft function.

## Introduction

Natural killer (NK) lymphoid cells are major components of innate immunity and serve as the first barrier against microbial infection and tumor development (1, 2). NK cells are cytolytic effector cells with two major modes of action. The first is direct lysis of human leukocyte antigen (HLA) class I-deficient target cells, a mode of action also called “spontaneous lysis.” Recognition of self-HLA class I molecules by inhibitory receptors known as killer cell immunoglobulin-like receptors (KIRs) present on NK cells inhibits their cytotoxicity and maintains self-recognition. In the case of the “missing self,” the absence of HLA class I molecules on target cells (i.e., tumor cells) prevents the inhibitory signal from triggering NK cell cytotoxicity (3). The second mode of action of NK cells is lysis induced by the interaction between the Fc receptor CD16 and the Fc fragment of an antibody (Ab) that recognizes foreign antigens on target cells (i.e., infected cells), an action also called antibody-dependent cellular cytotoxicity (ADCC) (4). In both cases, the lytic function of NK cells is dependent on cytolytic molecules, principally granzyme and perforin, and their activation leads to the production of various inflammatory cytokines, including interferon gamma (IFNγ).

In contrast to what was thought few decades ago (5, 6), it has now been clearly established that NK cells play a role in allograft rejections (7–9). The absence of self-major histocompatibility complex class I molecules on an allograft or the activation of the Fc receptor by donor-specific Abs induce NK cell activation, leading to direct cell lysis of the allograft and secretion of pro-inflammatory molecules that promote adaptive immunity (10–14). However, NK cells are two-faced. In addition to playing a role in allograft rejection, they are also involved in transplant tolerance according to studies in animal models (15–18). Allograft tolerance in solid organ transplantation [i.e., allograft acceptance in absence of immunosuppression (IS)] has been achieved in small animal models (19, 20), but translation to non-human primates and humans remains challenging. In clinical settings, protocols for tolerance induction have been attempted in kidney transplantation, mainly chimerism-based tolerance strategies (21), with some success (22–24). Tolerance has also been observed as a result of IS interruption due to non-compliance or medical decision [especially posttransplant lymphoproliferative disorders (PTLDs)] (24, 25). So-called spontaneously operationally tolerant kidney recipients display stable, good graft function for years. They are not immunosuppressed, they respond to immunological challenge (24, 26), and they do not harbor opportunistic infections (24, 25). From a clinical point of view, these patients do not differ from patients with stable graft function under standard IS (24, 25, 27).

This state of “spontaneous” tolerance is more frequently observed in liver transplantation in experimental models (28–30) and in humans, with no less than 80% recipients being tolerant after 10 years of transplantation (31). Interestingly, whereas mechanisms involved in the two situations appear to be different (31–36), compelling data in kidney and liver tolerant recipients have allowed identification of 63 NK-related genes that are specifically regulated in operationally tolerant kidney transplanted patients (32, 34). In the present study, we analyzed the NK cell phenotype and functional capacities from a cohort of patients operationally tolerant to a kidney graft [tolerant patient (TOL)] and compared the results to those from kidney transplanted patients with stable graft function [stable patient (STA)] under IS and healthy volunteers (HVs). We performed a deep phenotyping analysis of NK cells enriched by genotypic analysis of the KIR, perforin, and CD16 genes. In addition, we analyzed the capacity of NK cells to produce IFNγ and to degranulate and lyse target cells, using different assays mimicking the ADCC and spontaneous lysis. No differences were observed in the KIR, perforin, and CD16 genotypes between the three cohorts, but we found a dramatic decrease of perforin^+^ and NKp46^+^ NK cell frequencies, which was in turn associated with a decreased frequency of NK cells expressing the CD16 activator receptors in TOLs. These phenotypic changes are associated with broad impairment of both reverse ADCC and spontaneous lysis of NK cells from TOL, with a profound decrease of IFNγ^+^ and CD107a^+^ NK cells and chromium release in cytotoxicity assays. Conversely, despite impaired K562 cell lysis in the ^51^Cr release assay, we observed a normal reverse ADCC and an increased IFNγ^+^ and CD107a^+^ NK cells in the spontaneous lysis assay in STA recipients. These data highlight a defective NK cell profile in TOL recipients that may contribute to establishing a favorable microenvironment.

## Materials and Methods

### Operationally Tolerant Kidney Transplant Patients, STAs, and HVs

Healthy volunteer donors were recruited at the Blood Transfusion Center (EFS, Nantes, France). All subjects gave written informed consent in accordance with the Declaration of Helsinki. HVs were enrolled by the Etablissement Français du Sang (EFS, Nantes, France) within the context of a research contract. A convention has been signed between our laboratory (CRTI—INSERM UMR 1064) and the blood bank (Etablissement Français du Sang Pays de La Loire) and approval of an ethical committee was thus not necessary. The University Hospital Ethical Committee and the Committee for the Protection of Patients from Biological Risks approved the study for patients. The biological samples and data are gathered in accordance with French Law, more specifically with “Bioethical law” of August 6, 2004, Act no. 78-17 of January 6, 1978, on data processing, data, files, and individual liberties, with the European regulation: Directive 2004/23/EC of European Parliament and of the council of March 31, 2004 on setting, standards of quality and safety of donation, procurement, testing, processing, preservation, storage, and distribution of human tissue and cells, and with Directive 95/46/EC on the protection of individuals with regard to the processing of personal data and on the free movement of such data. PBMCs were isolated from blood by gradient centrifugation on Ficoll (Lymphoprep, Axis-Shield, PoC AS, Oslo, Norway). TOLs were defined as patients with stable kidney graft function (creatinemia <150 mmol/L and proteinuria <1 g/24 h) in the absence of IS for at least 1 year, and STAs were characterized by same functional criteria whereas under IS. Demographic and clinical data are presented in Tables 1 and 2. PBMCs of TOL used for the study were frozen cells coming from the DIVAT (*Données Informatisées et Validées en Transplantation*) biocoll (http://www.divat.fr/biocollection and CNIL number n°891735).

**Table 1:** Demographic and clinical characteristics from tolerant patient (TOL), stable patient (STA), and healthy volunteer (HV) used for phenotypic analysis and functional assay.

Demographic and clinical characteristics	TOL	STA	HV

	*n* = 11	*n* = 11	*n* = 15
Recipient gender (M/F)	6/5	6/5	10/5
Donor gender (M/F)	7/4	9/2	/
Recipient age at treatment	Median: 32 (range: 19–64)	Median: 49 (range: 35–77)	/
Recipient age at analysis	Median: 52 (range: 42–65)	Median: 52 (range: 37–78)	Median: 60 (range: 22–77)
Time post transplantation (years)	Median: 19 (range: 12–29)	Median: 2 (range: 1–6)	/
Donor age	Median: 27.5 (range: 6–41)	Median: 43 (range: 20–71)	/
Immunosuppression withdrawal duration	Median: 11 (range: 3–17)	/	/
Living/non-living donor	4/7	1/10	/
Human leukocyte antigen (HLA)-mismatch	5 at 0, 1 at 3, and 5 at 4	1 at 0, 1 at 1, 2 at 2, 2 at 3, 4 at 4, and 1 at 5	/
Anti-HLA (yes/no/unknown)	1/4/6	2/4/5	/
DSA (yes/no/unknown)	1/4/6	0/5/6	/
Cold ischemia duration (min)	1,245	983	/
Induction (yes/no/unknown)	7/4	10/1/0	/
Previous blood transfusion (yes/no/unknown)	9/1/1	3/6/2	/
CMV (yes/no/unknown)	6/3/2	3/3/5	/
EBV (yes/no/unknown)	7/2/2	4/1/6	/
Other infection (yes/no/unknown)	3/8/0	5/6/0	/
Cancer (yes/no/unknown)	2/9/0	1/10/0	/

**Table 2:** Demographic and clinical characteristics from tolerant patient (TOL), stable patient (STA), and healthy volunteers (HV) used for ^51^Cr release assay.

Demographic and clinical characteristics	TOL	STA	HV

	*n* = 7	*n* = 9	*n* = 8
Recipient sex (M/F)	5/2	5/4	4/4
Donor sex (M/F)	4/3	8/1	/
Recipient age at treatment	Median: 30 (range: 14–42)	Median: 41 (range: 23–59)	/
Recipient age at analysis	Median: 47 (range: 32–67)	Median: 49 (range: 43–66)	57.5 (range 29–62)
Time post transplantation (years)	Median: 23 (range: 6–31)	Median: 6 (range: 5–20)	/
Donor age	Median: 20 (range: 6–41)	Median: 67 (range: 10–67)	/
Immunosuppression withdrawal duration	Median: 9 (range: 1–18)	/	/
Living/nonliving donor	2/5	0/9	/
Human leukocyte antigen (HLA)-mismatch	3 at 0; 1 at 3, and 3 at 4	2 at 2; 5 at 4; 1 at 5, and 1 at 6	/
Anti-HLA (yes/no/unknown)	0/2/5	0/0/9	/
DSA (yes/no/unknown)	0/2/5	0/0/9	/
Cold ischemia duration (min)	1,245	1,739	/
Induction (yes/no/unknown)	6/1/0	7/2/0	/
Previous blood transfusion (yes/no/unknown)	5/0/2	4/1/4	/
CMV (yes/no/unknown)	2/1/4	1/0/8	/
EBV (yes/no/unknown)	5/1/1	0/0/9	/
Other infection (yes/no/unknown)	3/4/0	6/3/0	/
Cancer (yes/no/unknown)	1/6/0	3/6/0	/

### Cell Lines Culture

The P815 murine, the HLA class I-deficient 721.221 (221) and the K562 cell lines were cultured in RPMI 1640 medium (Life Technologies, Paisley, UK) containing glutamine (Life Technologies) and penicillin-streptomycin (LifeTechnologies). The P815 cell line medium was supplemented with 10% human serum (EFS, Nantes) and the 221 and K562 cell lines medium was supplemented with 10% Fetal Bovin Serum (Life Technologies).

### KIR and HLA Genotyping

Genomic DNA was extracted using a classical salting-out method (37). All DNAs were typed for KIR2DL1, 2DL2, 2DL3, 2DL4, 2DL5A/B, 3DL1, 3DL2, 3DL3, 2DS1, 2DS2, 2DS3, 2DS4/1D, 2DS5, and 3DS1, using a KIR multiplex polymerase chain reaction (PCR)-SSP method as previously described (38). Twenty-seven KIR locus-specific primers kindly provided by Pr David Senitzer (City of Hope, Duarte, CA, USA) for research purposesand split into four (G1, G2, G3, and G4) KIR primer-pair groups were used. The first G1 group amplify KIR2DL1, 2DS3, 2DL4, and 2DL2 loci; the second G2 group amplify 3DL1, 2DL3, 2DS2, and 3DS1 loci; the third G3 group amplify 2DS1, 3DL2, and 2DL5 loci; and the last G4 group amplify 2DS5, 3DL3, and 2DS4/1D loci. PCR amplifications were performed in 10 mL of reaction solution, including AmpliTaq Gold enzyme (0.75 U final, ThermoFischer Scientific, Villebon sur Yvette, France) and its bufferMgCl_2_ (2.25 mM final, ThermoFischer Scientific), dNTP mixtures (0.25 mM final each, ThermoFischer Scientific), G1/G2/G3/G4 primers (0.1–1.8 mM final), and DNA template (150 ng final). The multiplex PCR-SSP for KIR genotyping basic PCR protocol consists of one hold cycle at 93°C, 15 min followed by 32 cycles at 93°C, 20 s; 65°C, 30 s; 72°C, 30 s; and a final extension of 72°C, 5 min. PCR amplifications were performedeither in aGeneAmp 9700 (AppliedBiosystems/ThermoFischer Scientific), a T100 (Biorad, Les Ulis France) or a C1000 (Biorad) thermal cycler. The KIR PCR products were separated by electrophoresis on a homemade 3% agarose gel (Metaphor^®^, Ozyme, France) in 1X TBE (Sigma-Aldrich, Saint Quentin, France) buffer. KIR genes were identified depending on the length of each specific KIR amplification obtained in the four KIR primer-pair groups. High-resolution typing for HLA-A, HLA-B, and HLA-C loci (HLA laboratory, EFS Nantes, France) was carried out on samples by a Sequence Based Typing kit (Abbott Molecular Park, IL, USA). KIR genotypes were determined based on the presence or absence of activating KIR, KIR AA genotype presenting only KIR2DS4 as activating KIR, and KIR B^+^ genotype presenting several activating KIR. KIR ligand (i.e., A3/A11, Bw4, C1, and C2) were defined based on allelic HLA class I typing.

### Phenotype and Functional Assays by Flow Cytometry

Peripheral blood mononuclear cells were stained with Abs against CD3 (SK7), CD56 (NCAM16.2), CD16 (NKP15), CD8 (HIT8a), CD161 (DX12), ILT2 (GH1/75), CD57 (HNK-1), CD226 (DNAM-1) (DX11), NKp46 (9E2), granzyme A (CB9), perforin (γG9), NKG2D (1D11), CD4 (RPA-T4), CD8 (HIT8a), γ2TCR (B6), CD38 (HB7) (BD Biosciences), NKG2C (134591) (R&D Systems), NKp44 (Z231), NKp30 (Z25), NKG2A (Z199), KIR2DL1/S1 (EB6), KIR2DL2/3/2DS2 (GL183), KIR3DL1/S1 (Z27), HLA-DR (Immu357), KIR2DS4 (FES172), pan γδTCR (IMMU510) (Beckman Coulter, Fullerton, CA, USA), KIR2DL1 (143211) (R&D Systems), CD14 (RMO-52) (EFS, Rennes), KIR2DL2/3/2DS2 (4A8) (39), KIR2DL3/S2 (1F12) (39), and HLA-A,-B,-C (W6/32) (Biolegend). PBMCs were preincubated with anti-CD107a (H4A3; BD Biosciences, San Jose, CA, USA). NK cell degranulation was assessed after incubation for 5 h with media (negative control), with 721.221(221) (E: T ratio of 1:1), or with P815 cell line after a preincubation with CD16 specific mAb or IgG control at 10 µg/mL. For the last 4 h of incubation, the cells were treated with brefeldin A (Sigma) at 10 mg/mL to allow the intracellular accumulation of IFNγ. The cells were stained and permeabilized before intracellular IFNγ staining with PE-anti-human IFNγ (B27, BD Biosciences). Flow cytometry was performed using a FACSCalibur apparatus with CellQuest software (BD Biosciences) and analyzed using FlowJo 7.6.1 software (Tree Star, Ashland, OR, USA). The assay of PBMC incubation with P815 cell line and CD16 specific Ab was used to mimic the ADCC and interaction of the anti-CD16 Ab with the CD16 of NK cells, leading to their activation, IFNγ production and degranulation, which was measured by CD107a surface expression. Stimulation assay of PBMCs with the 221 cell line mimicked the direct NK cell activity, which was assessed by analyzing IFNγ production and CD107a surface expression.

### EVOS^®^ Fluorescence Cell Imaging

Natural killer cells were enriched after T-cell depletion of PBMCs using a CD3-specific Ab (BD Biosciences, San Jose, CA, USA) and murine IgG-coupled magnetic Dynabeads according to the manufacturer’s instructions (Dynal, Oslo, Norway). Enriched NK cells were activated with the 221 cell line as previously describes in the functional assay section. NK cells were stained with anti-NKp46 (9E2, BD Biosciences) and fixed with PFA 4%. Cells were then permeabilized in a PBS 0.1% saponin solution, and perforin intracellular expression was measured using anti-Perforin (Dg9, BD Biosciences) after 1 h incubation at room temperature. Samples were mounted with the ProLong^®^ Gold reagent (Invitrogen). After 48 h, cells were imaged using a ×60/0.075 NA oil immersion lens and acquired using phase contrast and fluorescence imaging by EVOS^®^ fluorescence microscope (ThermoFisher Scientific). Two light cubes were combined to identify NKp46-Alexa fluor 647 (RFP) and perforin-Alexa fluor 568 (Cy5) expressions.

### Perforin Gene Sequencing

Genomic DNA was analyzed to identify potential mutation on the perforin gene. Genomic DNA was isolated from peripheral blood using the standard phenol–chloroform protocol. We used the protocol described by Molleran Lee et al. (40) Briefly, exons 2 and 3 of the perforin gene were amplified by PCR using the following primers: for exon 2, 5′-CCCTTCCATGTGCCCTGATAATC-3′ and 5′-AAGCAGCCTCCAAGTTTGATTG-3′, and for exon 3, 5′-CCAGTCCTAGTTCTGCCCACTTAC-3′ and 5′-GAACCCCTTCAGTCCAAGCATAC-3′. For amplification, we used 500 ng of gDNA in 1× PCR buffer, 1.5 mmol/L of MgCl_2_, 0.2 mM of each dNTP, 0.4 µmol of each primer (forward and reverse), and 2.5 U of *Taq* pol (Invitrogen). Reaction conditions were 3 min at 95°C; 30 cycles of 45 s at 95°C, 30 s at 60°C, and 1 min 45 s at 72°C; and a last step of 10 min at 72°C. For the sequencing of the PCR product, we used the same primers as for DNA amplification for exon 2, and for exon 3 we used the same forward primer and two other reverse primers, one to better analyzed the 3′ end, 5′-TTGGTCTAATGGGAATACGAAG-3′ and one for the internal exon 3, 5′-CCATCACACCTCCATTAACGA-3′. DNA PCR products were sequenced using ABI BigDye terminator reactions and run on AB3730 capillary sequencer.

### ^51^Cr Release Assay

Cytotoxicity assay was performed in triplicate in a standard chromium release assay. K562 cells were labeled with 100 μCi Na^51^CrO_4_ (NEZ030, Perkin Elmer, Courtaboeuf, France) for 1 h at 37°C, and 1 × 10^3^ target cells were mixed with PBMCs at various effector/target ratios (100:1, 25:1, and 6.25:1). After 4 h at 37°C, 25 µL aliquots of supernatants were each mixed with 100 µL of scintillation liquid (OptiphaseSupermix, Wallack, United Kingdom) for measurement of radioactive content on a beta plate counter (Microbeta Jet 1450, PerkinElmer). The percentage of target cell lysis was calculated according to the following formula: [(experimental release − spontaneous release)/(maximum release − spontaneous release)] × 100. Maximum and spontaneous releases were, respectively, determined by adding 0.1% Triton X-100 or RPMI 1640 10% FBS on ^51^Cr-labeled K562 cells.

### Statistical Analysis

Statistical analyses were performed with Prism-6 software (GraphPad Software). The non-parametric Kruskal–Wallis test was used for comparisons of multiple groups followed by Dunn’s post-test to compare all paired of columns. Continuous non-parametric variables are expressed as medians (min and max). Non-parametric Spearman test was used for correlation analysis. Significance was defined as *p* less than 0.05. *p < 0.05, **p < 0.01, ***p < 0.001, and ****p < 0.0001.

## Results

### Circulating NK Cells from TOL Harbor a Defect in Expression of NKp46^+^ and CD16^+^ Activating Receptors and Perforin and Granzyme A Cytotoxic Molecules

**Figure 1:**
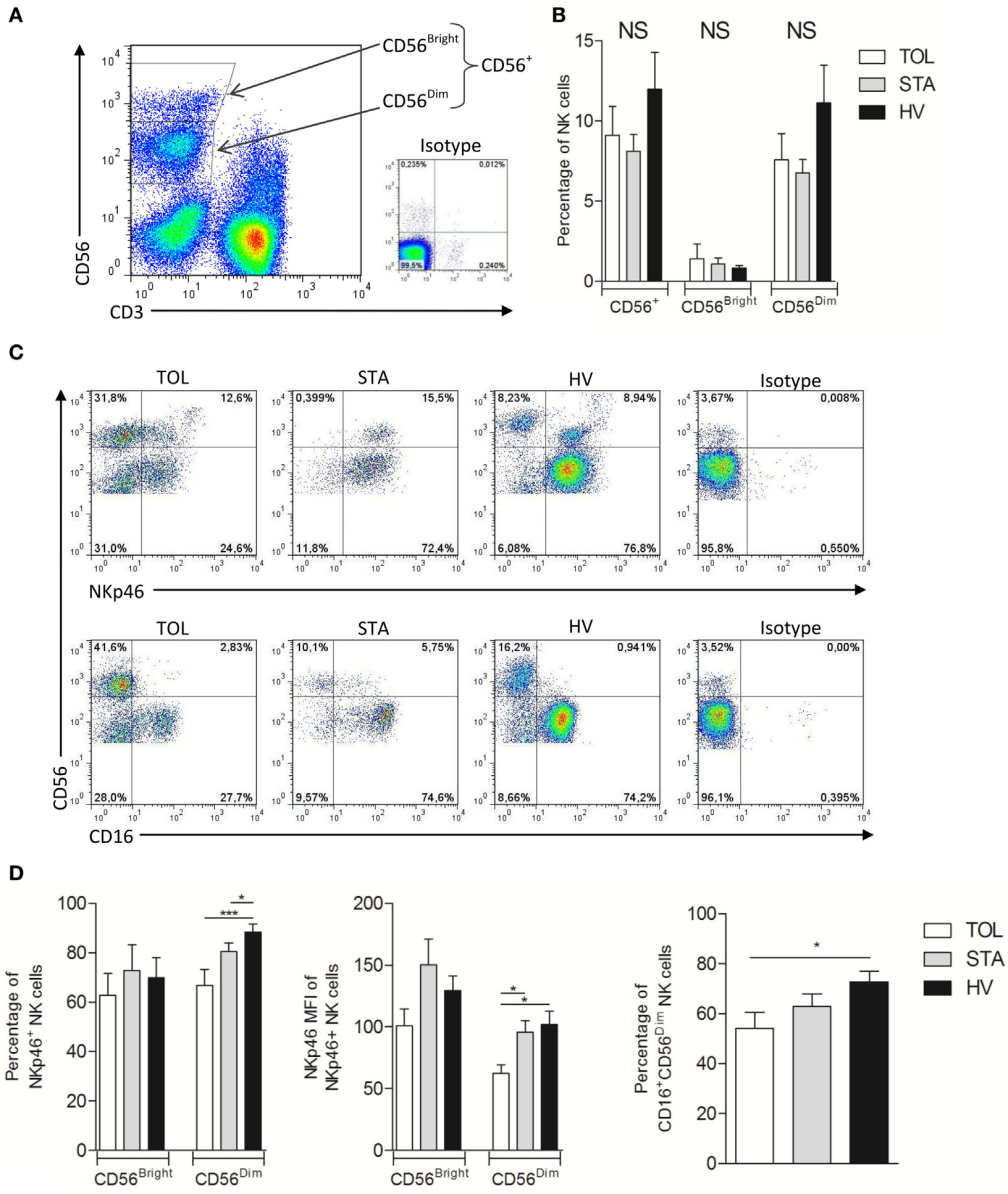
**(A)** Dotplot representation of the gates performed depending on CD3 and CD56 expression to divide natural killer (NK) cells into CD56^Bright^ and CD56^Dim^ subsets. Cells on the dotplot are lymphocytes gated regarding the morphology based on the SSC and FSC. Isotype controls are shown in the little dotplot. **(B)** Mean percentage with SEM of CD56^+^, CD56^Bright^, and CD56^Dim^ NK cells in tolerant patient (TOL) (white bar) (*n* = 11), stable patient (STA) (gray bar) (*n* = 11), and healthy volunteer (HV) (black bar) (*n* = 15). **(C)** Representative dotplot of NKp46 (up) and CD16 (down) expression in NK cells in TOL (left), STA (middle), HV (right), and isotype controls (far right). **(D)** Mean percentage with SEM of NKp46^+^ NK cells (left) and mean of MFI with SEM of NKp46 (middle) among CD56^Bright^ or CD56^Dim^ NK cells and mean percentage with SEM of CD16^+^CD56^Dim^ NK cells (right) in TOL (white bar) (*n* = 11), STA (gray bar) (*n* = 11), and HV (black bar) (*n* = 15). Significance was defined as *p < 0.05, **p < 0.01, ***p < 0.001, and ****p < 0.0001.

**Figure 2:**
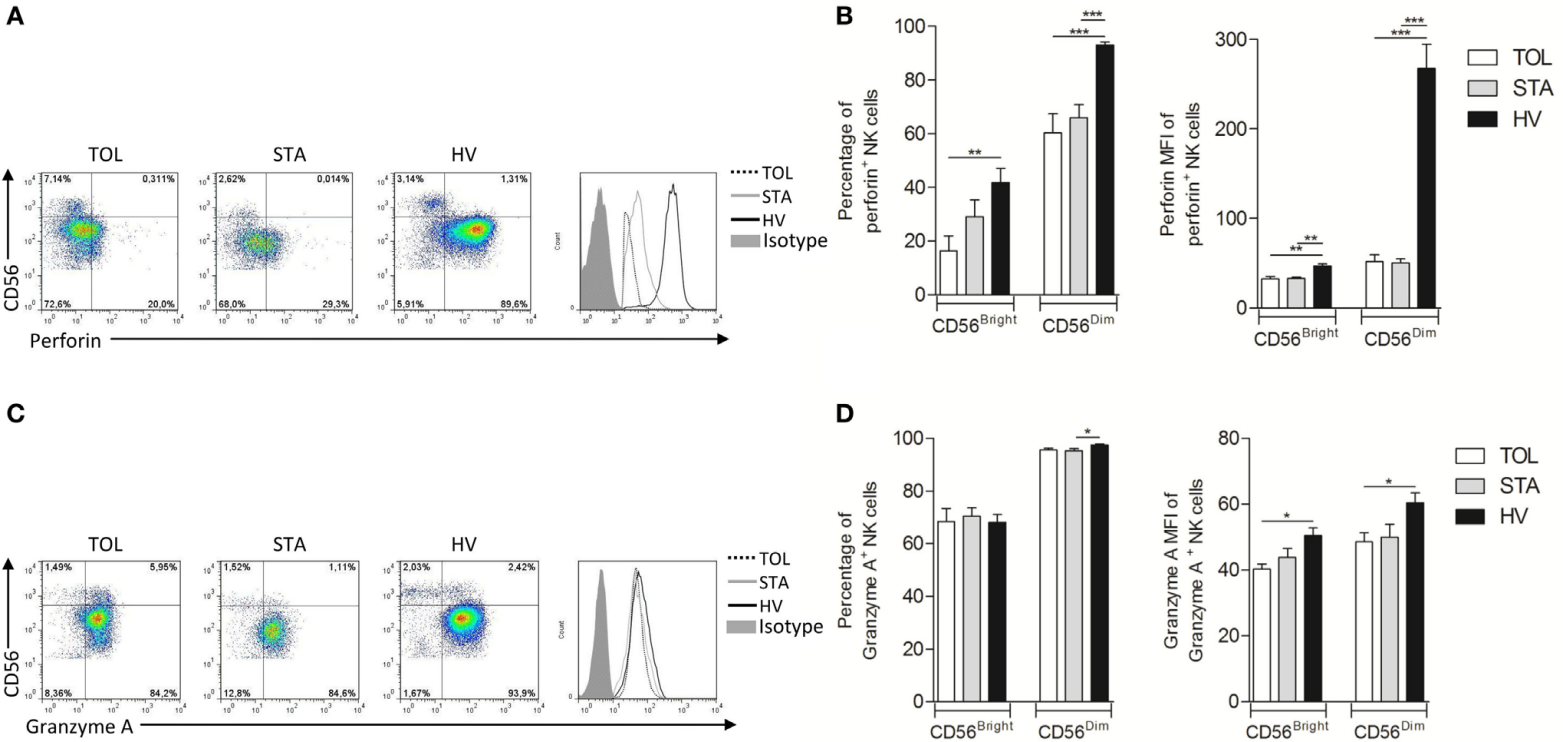
**(A)** Representative dotplot of perforin expression in tolerant patient (TOL) (left), STA (middle), and healthy volunteers (HV) (right); and histogram of perforin expression in CD56^Dim^ natural killer (NK) cells in TOL (dotted line), STA (gray line), HV (black line), and isotype control in the filled histogram. **(B)** Mean percentage with SEM of perforin^+^ NK cells and mean of MFI with SEM of perforin in NK cells in TOL (white bar) (*n* = 11), STA (gray bar) (*n* = 12), and HV (black bar) (*n* = 15) among CD56^Bright^ or CD56^Dim^. **(C)** Representative dotplot of granzyme A expression in TOL (left), STA (middle), and HV (right); and histogram of granzyme A expression in CD56^Dim^ NK cells in TOL (dotted line), STA (gray line), HV (black line), and isotype control in the filled histogram. **(D)** Mean percentage with SEM of granzyme A^+^ NK cells and mean of MFI with SEM of granzyme A in NK cells in TOL (white bar) (*n* = 11), STA (gray bar) (*n* = 12) and HV (Black bar) (*n* = 15) among CD56^Bright^ or CD56^Dim^. (Median and range are given in Table S1 in Supplementary Material.). Significance was defined as *p < 0.05, **p < 0.01, ***p < 0.001, and ****p < 0.0001.

We performed an exhaustive analysis of the phenotype of NK cells from TOL, STA, and HV. All analyses were conducted in CD3^−^CD56^+^ cells divided into two CD56^Bright^ (major producer of cytokines by NK) and CD56^Dim^ (mostly responsible of cytotoxicity by NK) subsets as shown in Figure 1A. This phenotypic analysis included activation receptors (NKG2D, NKG2C, NKp30, Nkp44, NKp46, 2B4, DNAM-1, CD16, CD161); inhibitory receptors (ILT2, KIR2DL1/S1, KIR2DL2/3/S2, NKG2A); and maturation (CD57), activation (CD38), and cytotoxic markers (perforin and granzyme A). Both TOL and STA displayed normal NK cell frequency compared to HV (Figure 1B) and we did not observe any significant difference the expression level of CD56. Interestingly, TOL had a significant decrease in the percentage of NKp46^+^CD56^Dim^ NK cells (*p* < 0.001 in TOL vs HV) associated with decreased expression of NKp46 compared with HV and STA (Figures 1C,D) (Table S1 in Supplementary Material). This defect is associated with a decrease of CD16^+^ NK cells in TOL (Figures 1C,D) (median and range are given in Table S1 in Supplementary Material). Granzyme and perforin are major instrumental molecules for cytotoxic NK cell activity. In steady-state conditions, CD56^Bright^ and CD56^Dim^ NK cells from TOL and STA displayed a significantly lower frequency of perforin^+^ NK cells associated with decreased expression *per se* in their granules (median and range are given in Table S1 in Supplementary Material) (Figures 2A,B) compared to HV. This pattern was associated with lower expression of granzyme A in CD56^Bright^ and CD56^Dim^ NK cell subsets (TOL vs HV, *p* < 0.05) (Figures 2C,D) (median and range are given in Table S1 in Supplementary Material). Altogether, these data suggest that despite a normal peripheral frequency, NK cells, particularly the CD56^Dim^ subset, from TOL have an impoverished profile, with decreased expression of major NK cell activator receptors. At steady state, NK cells from transplanted patients had dramatic lower levels of perforin and NK cells from TOL in particular displayed significantly lower levels of granzyme A but less significant than perforin.

### NK Cells from TOL Harbor a Cytotoxic Functional Impairment

**Figure 3:**
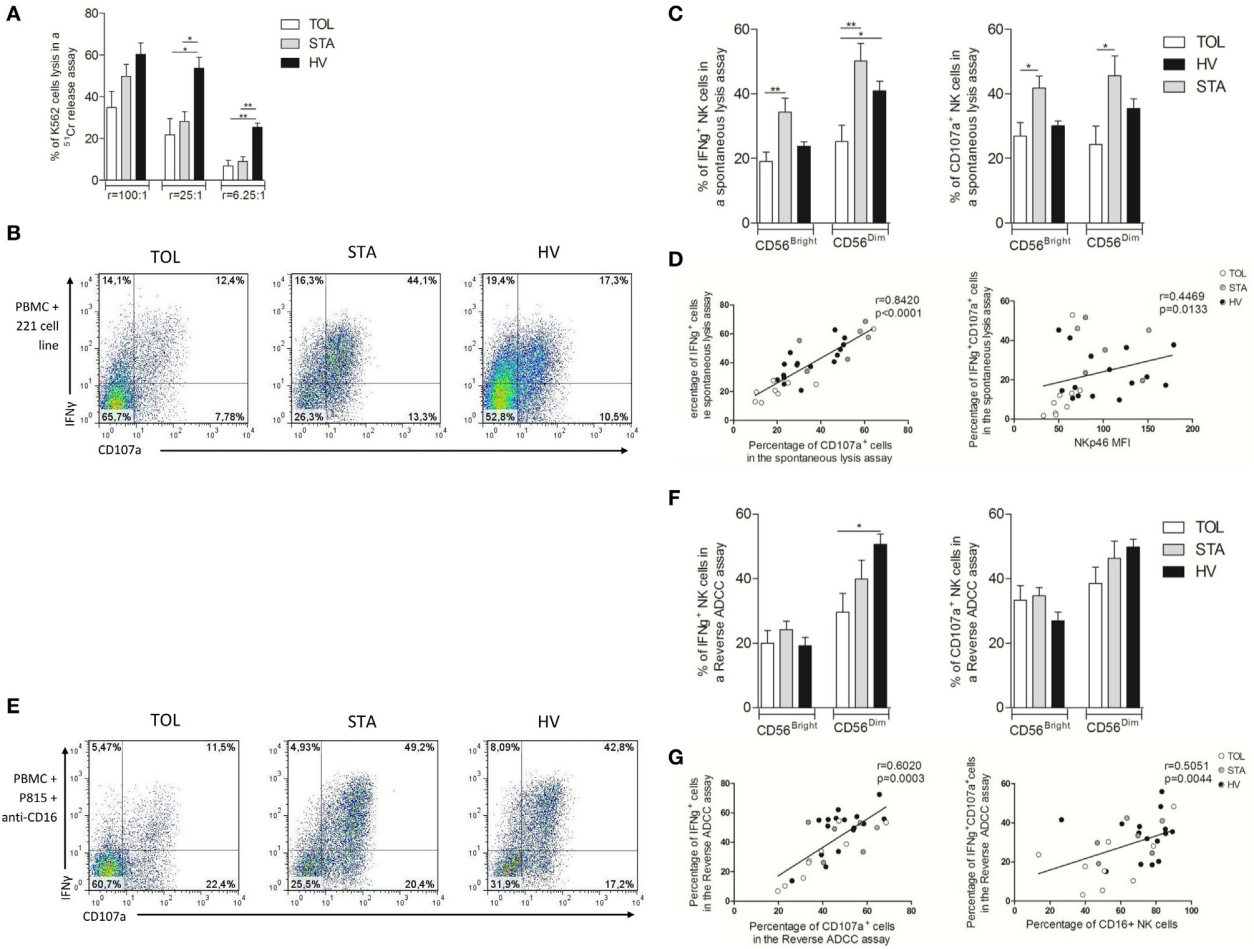
**(A)** Mean percentage with SEM of K562 lysis based on ^51^Cr release. Peripheral blood mononuclear cells (PBMCs) from tolerant patient (TOL), stable patient (STA), or healthy volunteer (HV) were cocultured with K562, and ^51^Cr release by K562 following lysis by PBMCs was evaluated. Three different PBMC:K562 ratios were analyzed: 100:1, 25:1, and 6.25:1. TOL (*n* = 7) are represented by white bars, STA (*n* = 9) by gray bars, and HV (*n* = 8) by black bars. (Median and range are given in Table S2 in Supplementary Material.) **(B)** Representative dot plots illustrating CD107a membranous expression and IFNγ production by CD56^Dim^ NK cells from one representative TOL, STA, and HV in a spontaneous lysis functional assay. **(C)** Mean percentage with SEM of IFNγ^+^ NK cells and the mean percentage with SEM of CD107a^+^ NK cells among CD56^Bright^ or CD56^Dim^ NK cells from TOL (white bars) (*n* = 9), STA (gray bars) (*n* = 7), and HV (black bars) (*n* = 15) following 5 h of coculture with 221 cell lines. **(D)** Correlation analysis between the percentage of IFNγ^+^ NK cells and the percentage of CD107a^+^ NK cells in the spontaneous lysis assay and correlation between the percentage of IFNγ^+^CD107a^+^ NK cells and the level of NKp46 (MFI) (*r* = Spearman correlation coefficient) (TOL = white dot, STA = gray dot, and HV = black dot). (Median and range are given in Table S2 in Supplementary Material.) **(E)** Representative dot plots illustrating CD107a membranous expression and IFNγ production by CD56^Dim^ NK cells from one representative TOL, STA, and HV in a reverse antibody-dependent cellular cytotoxicity (ADCC) assay. (Median and range are given in Table S2 in Supplementary Material.). **(F)** Mean percentage with SEM of IFNγ^+^ NK cells and the mean percentage with SEM of CD107a^+^ NK cells among CD56^Bright^ or CD56^Dim^ NK cells from TOL (white bars) (*n* = 9), STA (gray bars) (*n* = 7), and HV (black bars) (*n* = 15) following 5 h of coculture with P815 cell lines and anti-CD16. **(G)** Correlation analysis between the percentage of IFNγ^+^ NK cells and the percentage of CD107a^+^ NK cells in the reverse ADCC assay and correlation between the percentage of IFNγ^+^CD107a^+^ NK cells and the percentage of CD16^+^ NK cells (*r* = Spearman correlation coefficient) (TOL = white dot, STA = gray dot, and HV = black dot). Significance was defined as *p < 0.05, **p < 0.01, ***p < 0.001, and ****p < 0.0001.

**Figure 4:**
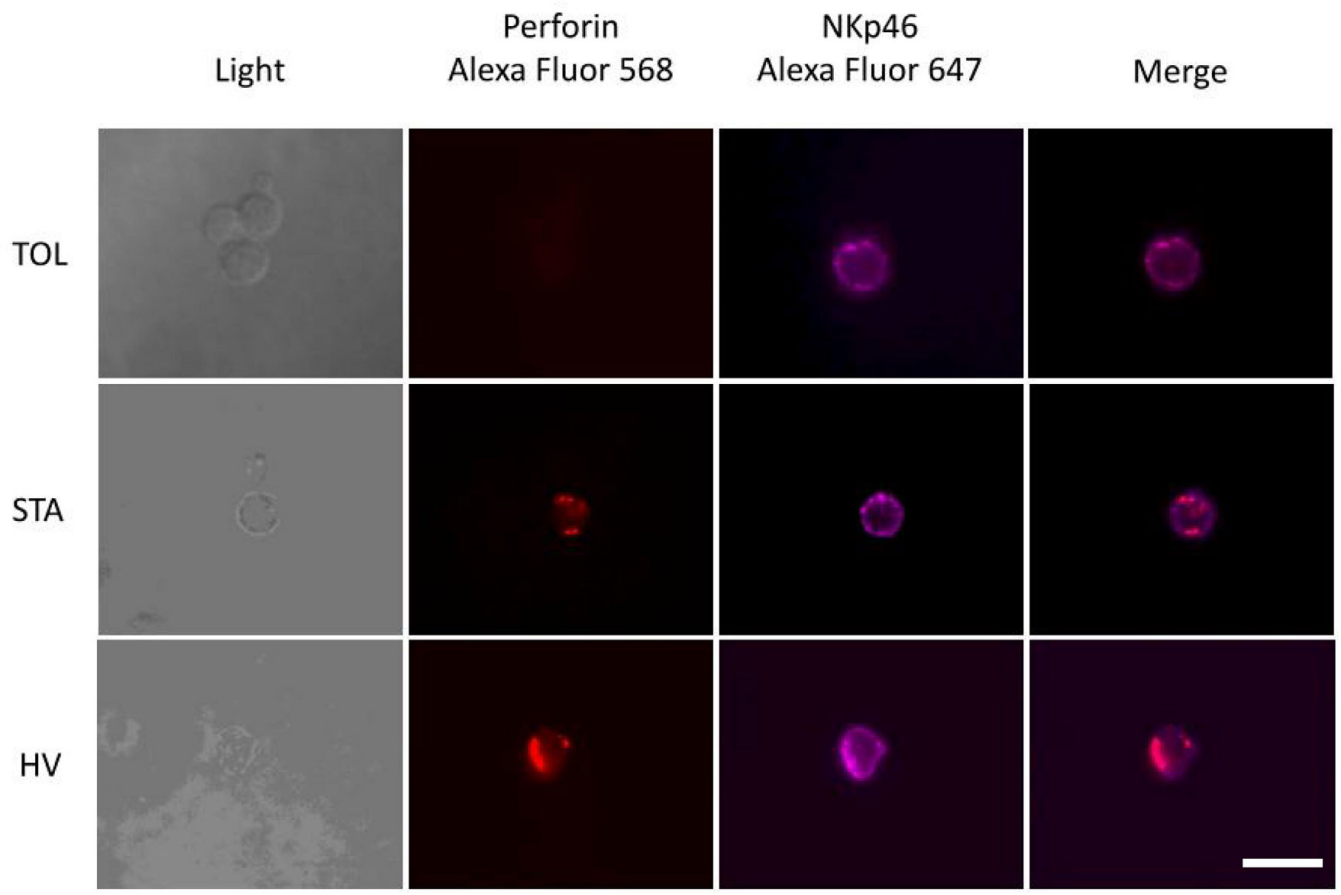
Microscopic analysis of perforin and NKp46 expression. Perforin and NKp46 expression were analyzed following 5 h coculture of peripheral blood mononuclear cells (PBMC) with 221 cell lines. Images were captured by using EVOS systems with light (*Left*) and fluorescent detectors perforin (AF568), NKp46 (Cy5), or an overlay of all fluorescent channels (*Right*). Examples are shown for one tolerant patient (TOL) (top), one stable patient (STA) (middle), and one healthy volunteer (HV) (bottom). NK cells were activated following the spontaneous lysis assay protocol and then labeled with anti-perforin-Alexa Fluor 568 and anti-NKp46-Alexa Fluor 647.

Because phenotypic analysis of NK cells from transplanted patients revealed important modifications with regard to activation of receptors and cytotoxic molecules, particularly for TOLs, we analyzed the functional capacities of these cells to determine if these phenotypic defects affect their function. Three different and complementary functional assays were performed to analyze the cytotoxic capacities of NK cells. The first was a functional assay based on ^51^Cr release by an HLA-Class I-negative K562 cell line following lysis by NK cells to analyze the “self-missing” process in the functional cytotoxic capacities of NK cells. We found a significant decrease in ^51^Cr released by K562 when cells are in contact with NK cells from TOL and STA compared to HV (Figure 3A). No difference was observed between TOL and STA, which suggested the same level of self-missing NK cell reactivity regardless of effector/target cell ratios (100:1, 25:1, and 6.25:1) (Figure 3A). We then used a second functional assay, in which peripheral blood mononuclear cells (PBMCs) were cultured with the 221 cell line lacking HLA class I expression. With this assay, we analyzed the direct lysis activity of NK cells by measuring IFNγ production, which indicated the activation status of NK cells and CD107a cell surface expression, which in turn reflected their degranulation process capacities. We found that IFNγ^+^CD56^Dim^ and CD107a^+^CD56^Dim^ NK cells were significantly decreased in TOL compared to STA and HV (*p* < 0.01 and *p* < 0.05, respectively, for IFNγ^+^, Table S2 in Supplementary Material and Figures 3B,C), whereas both IFNγ^+^CD56^Dim^, CD107a^+^CD56^Dim^ NK cells and IFNγ^+^CD56^bright^, CD107a^+^CD56^bright^ NK cells were increased in STA (Figures 3B,C). Interestingly, we observed a correlation between the percentage of IFNγ^+^ NK cells and the CD107a^+^ NK cells in the spontaneous lysis assay, suggesting that the cells that produced less IFNγ also had impairment in their degranulation process (Figure 3D). Moreover, we also observed a correlation between the expression of the NKp46 activating receptor and the level of NK cells IFNγ^+^CD107a^+^CD56^Dim^, which suggests that the NK cells impairment in the lysis process was due to the lower level of the activating receptor (Figure 3D). Finally, we performed a third test mimicking the lysis of target cells by ADCC in which we again measured IFNγ production and CD107a cell surface expression upon activation *via* CD16. In accordance with previous results, TOL had a decrease in IFNγ^+^CD56^Dim^ NK cells and CD107a^+^CD56^Dim^ NK cells (*P* < 0.05 in TOL vs HV for IFNγ) after anti-CD16 mAb stimulation, suggesting a deficit of activation and degranulation compared with NK cells from HV and STA (Table S2 in Supplementary Material; Figures 3E,F). As in the previous assay, we observed a correlation between IFNγ^+^ NK cells and CD107a^+^ NK cells, linking low cytokine production and a reduction in the degranulation process (Figure 3G). We also observed a correlation between the percentage of CD16^+^ NK cells and the percentage of IFNγ^+^CD107a^+^CD56^Dim^ NK cells (Figure 3G), suggesting that the lower percentage of CD16^+^ NK cells in TOL is partly responsible for the reversed ADCC impairment. Interestingly, analysis of IFNγ^+^CD107a^+^CD56^Dim^ NK cells after spontaneous lysis and reverse ADCC revealed a strong correlation between the two functional tests (*r* = 0.7087, *p* < 0.0001) (Figure S1 in Supplementary Material), meaning that patients who have impaired spontaneous lysis also have impaired reverse ADCC. Finally, we used an Invasive EVOS^®^ fluorescence cell imaging microscopic analysis, in which enriched NK cells from TOL, STA, or HV were cocultured for 5 h with the 221 cell line to measure intracellular perforin expression and NKp46 cell surface expression. We confirmed the very low level of perforin and NKp46 cell surface expression in NK cells from TOL after stimulation. By contrast, both were expressed in STA and HV (Figure 4). Altogether, the three complementary functional assays showed that NK cells from TOL display dramatically lower cytotoxicity through ADCC and mostly through the direct lysis and harbor a defect in IFNγ, perforin cells, and perforin/granzyme secretion at steady state and upon stimulation.

### NK Cell Function Impairment in TOL Is Not Associated with Modification of Their KIR Genetic Profile

**Figure 5:**
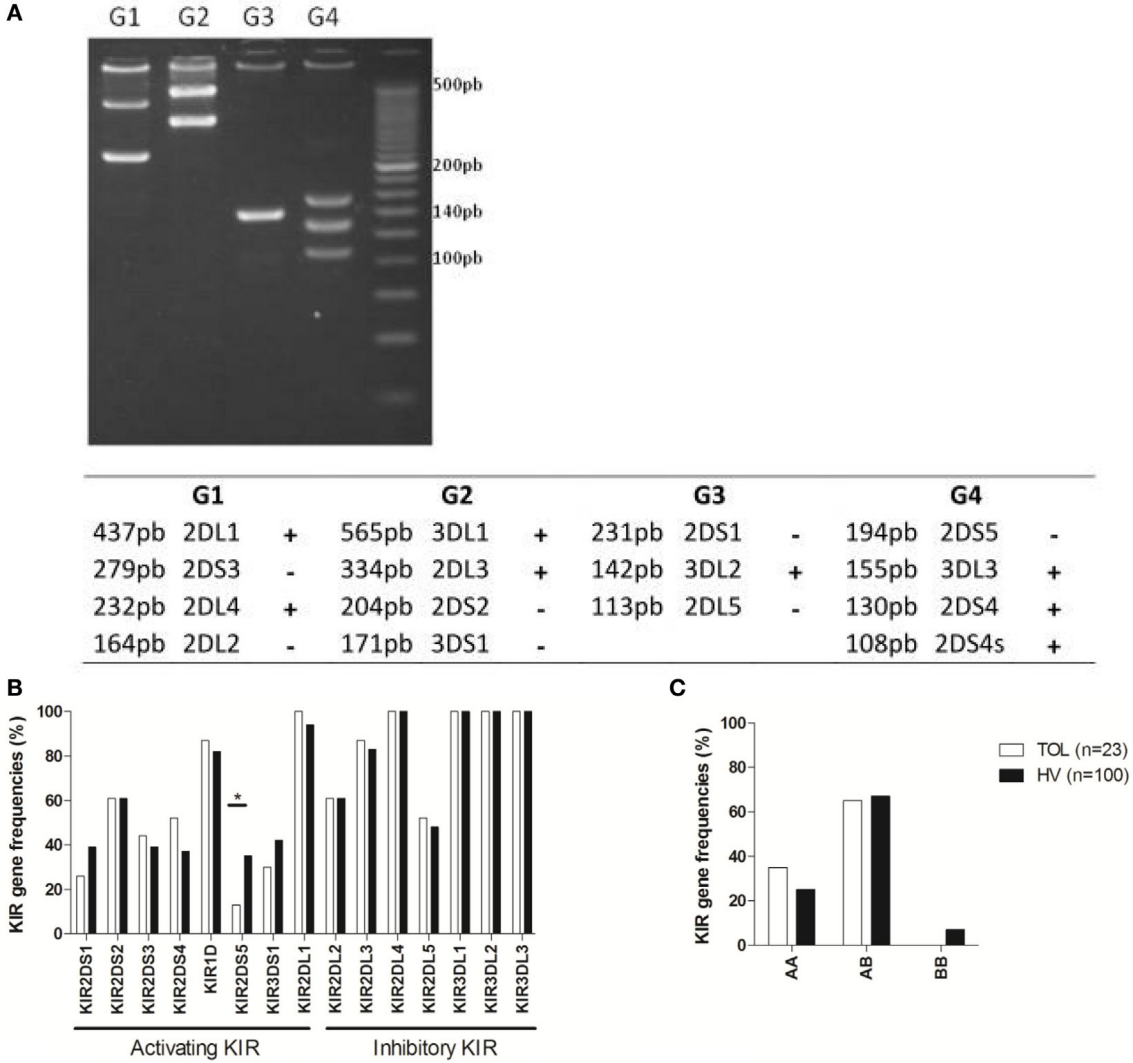
Representative 3% agarose gel electrophoresis of KIR multiplex polymerase chain reaction-SSP products and corresponding KIR amplification patterns and KIR profile in kidney transplanted patients. **(A)** The figure represents the presence (+) or absence (−) of each KIR gene in one representative tolerant patient (TOL) with the four groups of primers (G1, G2, G3, and G4) allowing the detection of all functional KIR genes. KIR primer-pair groups provide short amplicons from 108 to 565 bp. **(B)** Representation of the KIR genotype frequencies TOL (*n* = 23) and healthy volunteer (HV) (*n* = 100). **(C)** KIR genotype AA, AB, and BB frequencies in TOL (*n* = 23) and HV (*n* = 100). Significance was defined as *p < 0.05, **p < 0.01, ***p < 0.001, and ****p < 0.0001.

Killer cell immunoglobulin-like receptors are a family of receptors present on NK cells, which define their response according to their inhibitory and/or activating profiles. Because some genes are known to be differentially expressed at the transcriptional level in NK cells from TOL (27, 32, 34), we wondered if their KIR profile might also be associated with genetic modification. We analyzed both their KIR gene content and KIR gene frequencies by a multiplex PCR-SSP method (Table 3). Representative electrophoresis results of KIR multiplex PCR-SSP products and the corresponding KIR amplification patterns are shown in Figure 5A. KIR gene frequencies in TOLs were very similar to those in HV, with the only exception being a decreased frequency of the *KIR2DS5* gene in TOL (*p* = 0.046) (Figure 5B; Table 3), representing an activating KIR receptor for which the ligand is still unknown. No difference was observed for inhibitory KIR genes (Figure 5B). As for HV, TOL KIR genotyping mainly showed the AB KIR gene (Figure 5C). Flow cytometry analysis of activating and inhibitory KIR expression in NK cells did not highlight any difference of mean fluorescence intensity (MFI) or cell frequency between TOL and HV (data not shown). Altogether, these data indicate that NK cell function impairment in TOL is not associated with modification of the KIR genetic profile.

**Table 3:** Killer cell immunoglobulin-like receptor (KIR) genotyping results of TOLs cohort.

	KIR genotyping															
	2DL1	2DL2	2DL3	2DL4	2DL5	3DL1	3DL2	3DL3	2DS1	2DS2	2DS3	2DS4	1D	2DS5	3DS1	Genotypes KIR
TOL 1	+	−	+	+	−	+	+	+	−	−	−	−	+	−	−	AA
TOL 2	+	−	+	+	−	+	+	+	−	−	−	−	+	−	−	AA
TOL 3	+	−	+	+	−	+	+	+	−				+		−	AA
TOL 4	+	−	+	+	−	+	+	+	−	−	−	−	+	−	−	AA
TOL 5	+	−	+	+	−	+	+	+	−	−	−	+	+	−	−	AA
TOL 6	+	−	+	+	−	+	+	+	−	−	−	+	+	−	−	AA
TOL 7	+	−	+	+	−	+	+	+	−	−	−	+	+	−	−	AA
TOL 8	+	−	+	+	+	+	+	+	−	−	−	−	+	−	−	AA
TOL 9	+	+	+	+	−	+	+	+	−	+	−	−	+	−	−	AB
TOL 10	+	+	+	+	−	+	+	+	−	+	−	+	+	−	−	AB
TOL 11	+	+	+	+	−	+	+	+	−	+	−	+	+	−	−	AB
TOL 12	+	+	+	+	−	+	+	+	−	+	+	−	+	−	−	AB
TOL 13	+	+	−	+	+	+	+	+	−	+	+	+	−	−	−	AB
TOL 14	+	+	−	+	+	+	+	+	−	+	+	+	+	−	−	AB
TOL 15	+	+	−	+	+	+	+	+	−	+	+	+	+	−	−	AB
TOL 16	+	−	+	+	+	+	+	+	+	−	−	+	−	+	+	AB
TOL 17	+	+	+	+	+	+	+	+	+	+	+	−	+	−	−	AB
TOL 18	+	+	+	+	+	+	+	+	−	+	+	+	+	−	+	AB
TOL 19	+	+	+	+	+	+	+	+	−	+	+	+	+	−	+	AB
TOL 20	+	+	+	+	+	+	+	+	+	+	+	−	+	−	+	AB
TOL 21	+	+	+	+	+	+	+	+	+	+	+	−	+	−	+	AB
TOL 22	+	+	+	+	+	+	+	+	+	+	−	−	+	+	+	AB
TOL 23	+	+	+	+	+	+	+	+	+	+	+	+	−	+	+	AB

### TOL Do Not Harbor a Specific Perforin Genomic DNA Mutation Profile or CD16 Polymorphism

Defective perforin has been described in immune pathologies due to mutations in the perforin gene (41). Because of the strong decrease of perforin expression by NK cells from TOL at steady state and upon stimulation, we analyzed the perforin gene mutation levels following the amplification of exons 2 and 3 of the perforin gene in TOL, STA, and HV genomic DNA sequences. Classical and non-pathological mutations are present in the three groups at the same frequencies (C272 > T, C822 > T, C900 > T) (Table 4), suggesting no modification of the perforin genomic DNA phenotype in TOL that could be associated with their functional NK cell defect. Finally, because a CD16 polymorphism could influence ADCC as well as CD16 cell surface expression (42), we explored CD16 polymorphism in our patients. However, we found no particular differences among the three groups (data not shown).

**Table 4:** Mutations observed in the perforin gene from tolerant patient (TOL), stable patient (STA), and healthy volunteer (HV).

Perforin gene mutations	TOL	STA	HV
C272 > T	0/11	0/20	1/15
			1Het

C822 > T	1/11	4/20	4/15
	1Het	3 Het 1Homo	3 Het 1Homo

C900 > T	8/11	8/20	8/15
	7 Het 1Homo	6 Het 2Homo	4 Het 4Homo

## Discussion

The role of NK cells in organ transplantation is controversial. Although early studies suggested that they are not implicated in mice lacking B or T cells that retain NK cells and do not reject their graft (5), NK cells have also been identified in chronic allograft vasculopathy lesions (14, 43, 44) in Ab-mediated rejection (45–47) and rejection of bone marrow allografts in humans (48). We previously reported a specific transcriptional profile in the blood of patients tolerating a kidney graft that showed modulated expression of NK cell genes (32, 34). In the present study, we investigated their profile by analyzing the cells’ exhaustive activated/inhibitory phenotype, their functional cytotoxic capacities, KIR and CD16 genotypes, and extent of perforin gene mutation. The NK cell profile from TOL was compared with NK cells from STA and HV. This double comparison has two purposes. First, the absence of IS in the TOL and HV groups may not interfere with NK cell biology and function, and second, the comparison between TOL and STA allowed integrating the impact of the graft on NK cells and IS treatment on NK cells (49–51).

TOL patients harbor normal NK cell peripheral frequency and number (CD3^−^CD56^+^, CD56^Dim^, and CD56^Bright^ NK cell) in accordance with physiological levels of molecules associated with NK cell differentiation [CD57 (52), CD161 (53), NKG2A, KIR molecules (54)] (not shown). However, these patients harbor a defective profile of activation with a decreased frequency of activating *KIR2DS5* gene and NK cells that express NKp46 and CD16, suggesting that their activation is impaired. In comparison, STA also displayed a decreased frequency of NKp46^+^ NK cells, but they had normal CD16 expression. The lower expression level of these molecules, which play an important role in effector functions of NK cells, including both cell cytotoxicity and cytokine release (55–62), strongly suggests a defect in the functional activity of NK cells in TOL.

Natural killer cells activity is regulated by activating or inhibitory receptors and in accordance with their unique phenotype, we observed a strong impairment of the function of NK cells from TOL. Specifically, there was a profound decrease of IFNγ^+^ and CD107a^+^ NK cells in both ADCC and spontaneous lysis and a decrease of ^51^Cr release, which is according with decreased levels of the activating receptors, NKp46 and CD16. In association with the prominent defect in lysing K562 target cells and producing IFNγ upon stimulation, NK cells from TOL dramatically lacked intracellular perforin and harbored lower expression of granzyme A. By contrast, whereas NK cells from STA also had lower ^51^Cr release, they displayed a normal ADCC and even had higher spontaneous lysis compared to HV.

A key question is why NK cells from TOL patients express lower amounts of these molecules. Although their levels of expression may vary with age (63), age was not a confounding factor in this study (Table 1). The lower CD16 expression does not correspond with any particular CD16 polymorphism in TOL that could explain this lower ADCC activity. Similarly, whereas KIR polymorphism is associated with various infections, autoimmune diseases, and cancers and has a major role in the structure and the function of NK cells (64), genetic analysis did not reveal any major differential KIR expression on the surface of NK cells from TOL that could explain their impairment. Finally, the defect of perforin of NK from TOL is not due to an abnormal mutation level of the perforin gene, which is not surprising given the absence of associated related pathologies in these patients (41). We suggest that this low perforin level is due to transduction or translation regulation *via* external factors that could also act on granzyme A, CD16, and NKp46. Surprisingly, we also found a lack of intracellular perforin in STA at steady state despite normal ADCC and spontaneous lysis. Since it is known that IS dramatically influences NK cell function (49–51), we hypothesize an effect of IS. Indeed, after stimulation and culture in IS-free medium (Invasive EVOS^®^ fluorescence cell imaging microscopic analysis) when perforin production is no longer inhibited by the treatment, we clearly confirm the absence of perforin only in NK cells from TOL, whereas it is strongly expressed in STA. The high level of IFNγ^+^ NK cells and the recovery of perforin expression in NK cells following the spontaneous lysis assay strongly suggests that the impairment of NK cell cytotoxicity in STA patients in the ^51^Cr assay is not IFNγ or perforin dependent and implicates other pathways.

It has been shown that NKp46^+^ and CD16^+^ receptors could be downregulated upon stimulation (65–67). Indeed, in acute myeloid leukemia (AML) it is known that AML cells induce NK cells abnormalities, including the CD16 and NKp46 activating receptor downregulation but also the apoptosis (68, 69). In addition, low expression of NKp46 has been reported as an “evasion mechanism” associated with low cell activity in cancer (70), mainly following chronic stimulation (71), or described as an “exhausted” profile in chronically infected patients (72, 73), patients with cancer (Kaposi sarcoma, PTLD) (74, 75), a mechanism involved in peripheral tolerance. In the same way, chronic exposure to allo-reactive donor antigens from the transplant strongly stimulates NK cells and induces downregulation of these receptors. We hypothesize that the low NKp46 expression likely participates in a “pro-tolerant milieu” as previously reported (76–78), with establishment of a non-deleterious environment or “active shut-down process” to avoid excessive response in these patients following environmental events.

Thus, in the last decade, the status of NK cells has gone from “detrimental role” to “no role” and then to “beneficial role” in the field of transplantation. In animal models as in humans, NK cells have, thus, been shown to have beneficial effects and may be potent regulators of immunity. They delay allograft rejection by downregulating homeostatic CD8^+^ T-cell proliferation by competing for IL15 (15, 79), and they are able to inhibit clonal expansion of antigen-stimulated T cells (15, 80, 81) in addition to killing dendritic cells (17, 18, 82). Thus, NK cells clearly influence graft outcome and one challenge is to reconcile their role with their potential tolerizing or facilitating role in transplantation. Finally, NK cells are engaged in crosstalk with different immune cells, such as monocytes (83, 84), B cells (85), and Treg cells (86, 87) *via* mechanisms involving TGFβ (86) or contact dependency (88). In a same way, NK cell degenerative functions are mediated by IL21 derived from autoreactive CD4^+^ T cells (89). Moreover, a link between NK cells and increased induction of Tregs has been clearly established (90, 91) in correlation with higher TGFβ level in inflammatory situations (92). This general “regulatory” profile fits with the NK cell profile of TOL that correlates with increased frequency of Treg cells with higher immunosuppressive properties (93), higher numbers of granzyme B regulatory B cells (94), and regulation by IL21 secretion by CD4^+^ T cells (94) in a TGFβ environment (95). Finally, these data agree with recent data showing a clear link between NK cytotoxicity and DSA since these patients rarely develop donor-specific Ab (24, 27).

We hypothesize that this “favorable” or “shut-down” environment contribute pro-tolerogenic conditions, where each cell may play a role. How these cells interact, orchestrate, and/or control each other and their capacity to maintain homeostatic environment remain to explore. Despite this profound defect NK cells, which constitute the first line of innate and adaptive defense against infection and tumors, TOL do not experience more infections than healthy individuals and are able to respond normally to stimulation such as vaccination (25). This suggests that this profile does not affect their innate immunity, and it is likely strongly regulated to maintain “healthy homeostasis” in these patients. This study paves the way for dissecting more deeply the interplay of these immune cells. These observations may have critical implications for the discovery of new effective tolerance strategies to monitor NK cells in transplantation.

## Ethics Statement

Healthy volunteer (HV) donors were recruited at the Blood Transfusion Center (EFS, Nantes, France). All subjects gave written informed consent in accordance with the Declaration of Helsinki. HVs were enrolled by the Etablissement Français du Sang (EFS, Nantes, France) within the context of a research contract. A convention has been signed between our laboratory (CRTI—INSERM UMR 1064) and the blood bank (Etablissement Français du Sang Pays de La Loire) and approval of an ethical committee was, thus, not necessary. The University Hospital Ethical Committee and the Committee for the Protection of Patients from Biological Risks approved the study for patients. The biological samples and data are gathered in accordance with French Law, more specifically with “Bioethical law” of August 6, 2004, Act no. 78-17 of January 6, 1978, on data processing, data, files, and individual liberties, with the European regulation: Directive 2004/23/EC of European Parliament and of the council of March 31, 2004 on setting, standards of quality and safety of donation, procurement, testing, processing, preservation, storage, and distribution of human tissue and cells, and with Directive 95/46/EC on the protection of individuals with regard to the processing of personal data and on the free movement of such data.

## Author Contributions

SB and CR designed the study; ED, GD, RO, J-PJ, and CP acquired data; MC, PG, MG, and J-PS collected and provided important samples; ED, RD, KG, ND, PP, CP, SC, NG, CR, and SB analyzed and interpreted data; ED, CR, and SB wrote the manuscript; and all authors approved the final version of the manuscript.

## Conflict of Interest Statement

The authors declare that the research was conducted in the absence of any commercial or financial relationships that could be construed as a potential conflict of interest.

## Abbreviations

^51^Cr, chromium 51; ADCC, antibody-dependent cellular cytotoxicity; APC, antigen-presenting cell; CNI, calcineurin inhibitor; DSA, donor-specific antibody; HLA, human leukocyte antigen; HV, healthy volunteer; IFNγ, interferon gamma; IS, immunosuppression; KIR, killer cell immunoglobulin-like receptors; MFI, mean fluorescence intensity; MHC, major histocompatibility complex; NK, natural killer; PBMC, peripheral blood mononuclear cell; PCR, polymerase chain reaction; PTLD, posttransplant lymphoproliferative disorder; STA, stable patient; SSP, sequence specific primer; TOL, tolerant patient; Tregs, regulatory T cells.
