# Corrigendum: Broad Impairment of Natural Killer Cells From Operationally Tolerant Kidney Transplanted Patients

**DOI:** 10.3389/fimmu.2018.00589

**Published:** 2018-03-26

**Authors:** Emilie Dugast, Gaëlle David, Romain Oger, Richard Danger, Jean-Paul Judor, Katia Gagne, Mélanie Chesneau, Nicolas Degauque, Jean-Paul Soulillou, Pascale Paul, Christophe Picard, Pierrick Guerif, Sophie Conchon, Magali Giral, Nadine Gervois, Christelle Retière, Sophie Brouard

**Affiliations:** ^1^Centre de Recherche en Transplantation et Immunologie UMR1064, INSERM, Université de Nantes, Nantes, France; ^2^Institut de Transplantation Urologie Néphrologie (ITUN), CHU Nantes, Nantes, France; ^3^Etablissement Français du sang, Nantes, France; ^4^CRCINA, INSERM, Université d’Angers, Université de Nantes, Nantes, France; ^5^LabEx Transplantex, Université de Strasbourg, France; ^6^Nephrology Dialysis Renal Transplantation Center, Assistance Publique des Hôpitaux de Marseille, Hospital de la Conception, UMR 1076, Vascular Research Center of Marseille, INSERM, Aix-Marseille University, Marseille, France; ^7^Établissement Français du Sang Alpes Méditerranée, Marseille, France; ^8^ADES UMR 7268, CNRS, EFS, Aix-Marseille Université, Marseille, France; ^9^CIC Biotherapy, CHU Nantes, Nantes, France

**Keywords:** natural killer, cytotoxicity, tolerance, kidney, transplantation

There was a mistake in the authorship. The name of Jean-Paul Soulillou was unintentionally omitted. Dr. Soulillou has contributed to collecting and providing important biological samples used for the manuscript, and as such he should be included in the authorship. The authors apologize for the mistake. This error does not change the scientific conclusions of the article in any way.

With the inclusion of Dr. Soulillou’s name in the authorship, the paragraph of Author Contributions should also be corrected as follows:

SB and CR designed the study; ED, GD, RO, J-PJ, and CP acquired data; MC, PG, MG, and J-PS collected and provided important samples; ED, RD, KG, ND, PP, CP, SC, NG, CR, and SB analyzed and interpreted data; ED, CR, and SB wrote the manuscript; and all authors approved the final version of the manuscript.

This correction does not change the scientific conclusions of the article in any way.

The authors apologize for the mistake.

The original article has been updated.

## Conflict of Interest Statement

The authors declare that the research was conducted in the absence of any commercial or financial relationships that could be construed as a potential conflict of interest.

