# Supplementary material for: Broad Impairment of Natural Killer Cells From Operationally Tolerant Kidney Transplanted Patients
Source: Front Immunol. 2017 Dec 11;8:1721. doi: 10.3389/fimmu.2017.01721 (PMC5732263; doi:10.3389/fimmu.2017.01721)
Supplement: Supplementary file 1 [file table_1.pdf]

Phenotyping			
	TOL	STA	HV
Percentage of CD3$^-$CD56$^+$	7.46 [1.57;22.2]	8.39 [2.37;15.5]	9.37 [4.62;41]
Percentage of CD3$^-$CD56^{Bright}	0.513 [0.135;10.4]	0.631 [0.162;4;98]	0.662 [0.234;2.08]
Percentage of CD3$^-$CD56^{Dim}	6.49 [1.35;21.8]	7.9 [1.87;10]	8.67 [3.83;40.4]
Percentage of CD3$^-$CD56^{Bright} NKp30$^+$	23.05 [10.9;49.2]	40.2 [29.4;51]	25.3 [17.9;46.1]
Percentage of CD3$^-$CD56^{Dim} NKp30$^+$	29.1 [9.86;42.9]	51.05 [41.1;61]	47.1 [28;66.2]
Percentage of CD3$^-$CD56^{Bright} NKp46$^+$	58.1 [26;98.1]	94.8 [19.3;99.1]	82.5 [18.1;99.9]
Percentage of CD3$^-$CD56^{Dim} NKp46$^+$	76.8 [25.1;91.2]	82.8 [50.9;92.3]	91.7 [44;96.6]
NKP46 MFI of CD3$^-$CD56^{Bright} NKp46$^+$	96.6 [41.4;173]	130 [77.8;311]	121 [73.9;208]
NKP46 MFI of CD3$^-$CD56^{Dim} NKp46$^+$	59.4 [33;116]	80.2 [59.9;151]	88.1 [50.2;179]
Percentage of CD3$^-$CD56^{Bright} CD16$^+$	16.8 [0.143;50]	26.8 [0.136;51.4]	7.34 [0.585;24.9]
Percentage of CD3$^-$CD56^{Dim} CD16$^+$	50.8 [13.5;90]	63.5 [39.9;88.7]	77.8 [26.5;89.3]
Percentage of CD3-CD56^{Bright} granzyme A+	72.94 [42.9;89.6]	72.05 [50.75;88.8]	68.6 [49.7;87.2]
Percentage of CD3-CD56^{Dim} granzyme A+	96.5 [90.3;99.6]	95.6 [89.2;98.7]	97.9 [95.04;99.22]
Percentage of CD3-CD56^{Bright} perforin+	8.208 [2.855;53.1]	21.98 [5.607;69.3]	41.8 [4.658;72.5]
Percentage of CD3-CD56^{Dim} perforin+	64.9 [24;95.91]	67.5 [40.81;89.78]	94.6 [84.5;98.45]
Granzyme A MFI of CD3-CD56^{Bright} granzyme A+	40.9 [32.8;46.3]	43.15 [32.8;60.3]	51.6 [33;64]
Granzyme A MFI of CD3-CD56^{Dim} granzyme A+	46.1 [36;65.6]	51.4 [32.1;74.9]	63.7 [37.5;75.9]
Perforin MFI of CD3-CD56^{Bright} perforin+	30.6 [23.2;50.7]	31.85 [24.4;43.4]	46.8 [30.30;63.6]
Perforin MFI of CD3-CD56^{Dim} perforin+	42.6 [29.4;99.3]	48.9 [31.6;83.3]	276 [71.8;447]

Supplemental table 1: Median and range from the phenotypic analysis performed on TOL, STA and HV.
