# Supplementary material for: Broad Impairment of Natural Killer Cells From Operationally Tolerant Kidney Transplanted Patients
Source: Front Immunol. 2017 Dec 11;8:1721. doi: 10.3389/fimmu.2017.01721 (PMC5732263; doi:10.3389/fimmu.2017.01721)
Supplement: Supplementary file 2 [file table_2.pdf]

| Functionnal assay | | | |
|---|---|---|---|
| **Reverse ADCC** | TOL | STA | HV |
| Percentage of $CD3^-CD56^{Bright}CD107a^+$ | 31.4 [18;58.5] | 31.9 [26.5;47.3] | 21 [11.9;43.9] |
| Percentage of $CD3^-CD56^{Dim}CD107a^+$ | 33.9 [19.7;68.6] | 45.3 [26.1;64.5] | 47.1 [38.3;67.7] |
| Percentage of $CD3^-CD56^{Bright}IFN\gamma^+$ | 17.6 [7.14;41.6] | 24.7 [13.5;35.3] | 16.7 [7.21;44.4] |
| Percentage of $CD3^-CD56^{Dim}IFN\gamma^+$ | 27.3 [6.91;54.5] | 49.1 [13.8;53.6] | 55 [23.3;72.4] |
| | | | |
| **Spontaneous lysis** | TOL | STA | HV |
| Percentage of $CD3^-CD56^{Bright}CD107a^+$ | 26.5 [8.7;45.6] | 42.4 [24.6;55.2] | 30.5 [16.2;38.2] |
| Percentage of $CD3^-CD56^{Dim}CD107a^+$ | 19.1 [9.97;64.2] | 52.3 [23;61.8] | 31 [20.1;50.9] |
| Percentage of $CD3^-CD56^{Bright}IFN\gamma^+$ | 16.9 [6.94;33.4] | 36.2 [22.2;55] | 23.8 [15.3;33.5] |
| Percentage of $CD3^-CD56^{Dim}IFN\gamma^+$ | 20.7 [12.4;63.4] | 55.4 [31.4;68.6] | 39.5 [20.9;62.8] |
| | | | |
| **$^{51}$Cr release** | TOL | STA | HV |
| Percentage of K562 cell lysis ratio 100:1 | 38.74 [10.5;67.7] | 52.54 [23.1;80.7] | 56.66 [38.13;80.6] |
| Percentage of K562 cell lysis ratio 25:1 | 23.79 [0.9;61.7] | 25.6 [7.7;45.99] | 53.04 [30.96;78.4] |
| Percentage of K562 cell lysis ratio 6.25:1 | 6.391 [0;21] | 8.1 [1.8;20.26] | 25.04 [16.27;34.21] |

**Supplemental table 2:** Median and range from the functional assays performed on TOL, STA and HV.
