# Supplementary figures and images for: Broad Impairment of Natural Killer Cells From Operationally Tolerant Kidney Transplanted Patients
Source: Front Immunol. 2017 Dec 11;8:1721. doi: 10.3389/fimmu.2017.01721 (PMC5732263; doi:10.3389/fimmu.2017.01721)

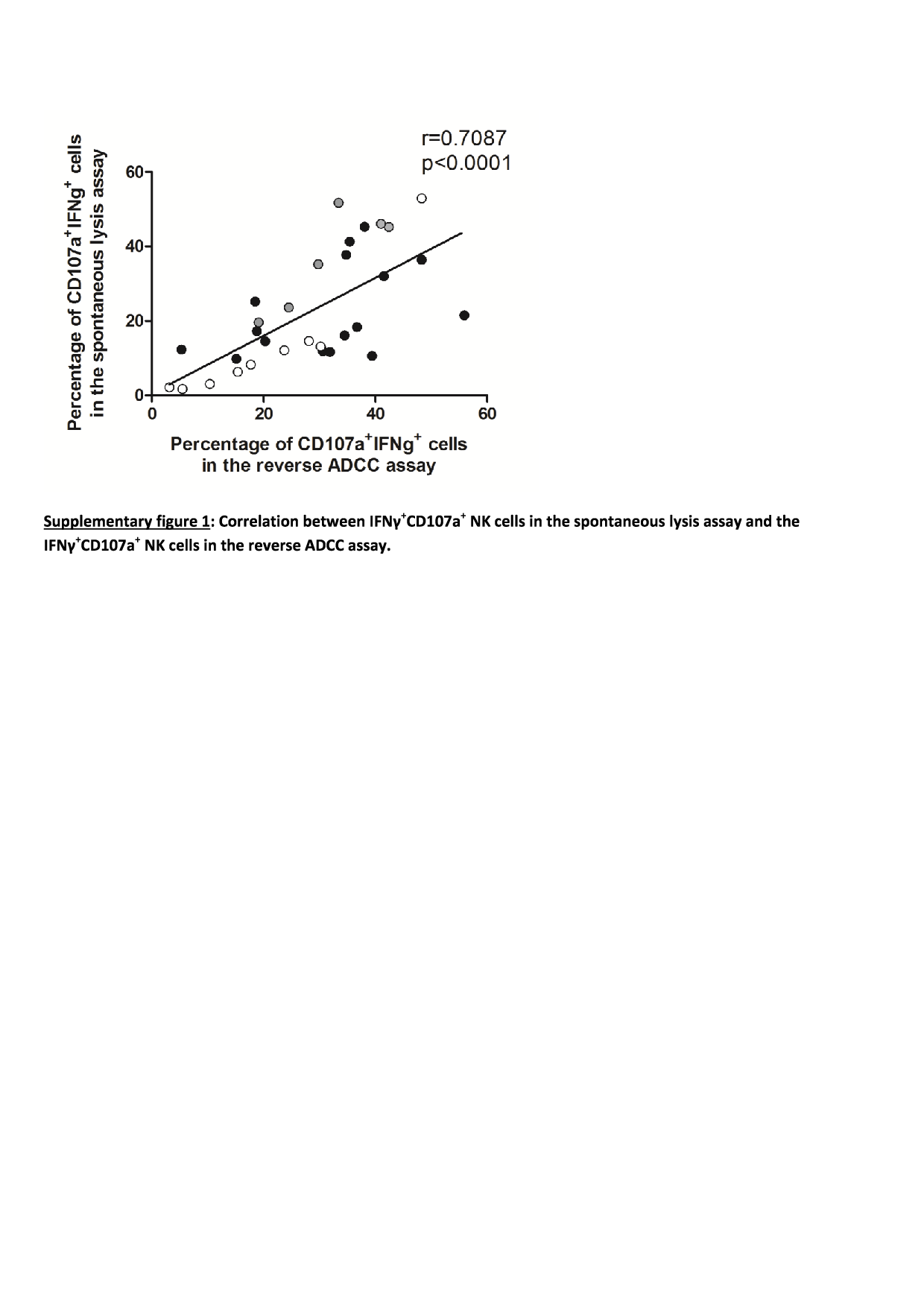

Supplement: Supplementary file 3 [file image_1.tif]
